# Role of amino acid metabolism in osteoporosis: Effects on the bone microenvironment and treatment strategies (Review)

**DOI:** 10.3892/mmr.2025.13577

**Published:** 2025-05-26

**Authors:** Chang Zhou, Jiaheng Zhang, Qizhi Liu, Yanghongxu Guo, Mengyuan Li, Jing Tao, Sujuan Peng, Ronghui Li, Xianguang Deng, Guomin Zhang, Huiping Liu

**Affiliations:** College of Integrative Medicine, Hunan University of Traditional Chinese Medicine, Changsha, Hunan 410208, P.R. China

**Keywords:** osteoporosis, amino acid metabolism, osteoclast, osteoblast, bone marrow mesenchymal stem cell

## Abstract

Osteoporosis is a metabolic disease characterized by an imbalance in bone remodeling. Its pathogenesis involves a functional imbalance of osteoblasts, osteoclasts and bone marrow mesenchymal stem cells. Amino acid metabolism is a key biochemical process for maintaining biological activities, including protein synthesis, energy supply and signal transduction. Amino acid metabolism affects bone homeostasis by regulating osteocyte function and the bone microenvironment. Branched-chain amino acid and aromatic amino acid metabolism are involved in the regulation of bone mineral density. The present review demonstrates the mechanism of amino acid metabolism in osteoporosis and its potential therapeutic value. In addition, the present review aimed to summarize the application of Mendelian randomization and metabolomic methods to provide a reference for future research and clinical interventions.

## 1. Introduction

Continuous bone formation and absorption are key processes in maintaining bone health. In adolescence, the rate of novel bone formation in the body is higher than that of old bone degradation, and thus, bone mass increases. However, after the age of 20 years, this process slows and the majority of individuals reach peak bone mass at the age of 30 years (1,2). Osteoblasts and osteoclasts serve key roles in bone remodeling in the bone microenvironment. The balanced regulation of osteogenic and adipogenic differentiation of bone marrow mesenchymal stem cells is involved in the formation of novel bone mass (3); these are the primary components of the bone microenvironment. Osteoporosis is a chronic systemic endocrine and metabolic disorder. Primary osteoporosis caused by aging or sex hormone deficiency, and secondary osteoporosis caused by hyperthyroidism, diabetes, obesity, Cushing's syndrome, anorexia, rheumatoid arthritis (RA) or adverse drug effects have similar potential mechanisms, namely, an imbalance of bone remodeling such that the loss of bone mass exceeds the formation of new bone (4). Low bone mineral density (LBMD), including osteoporosis and low bone mass, has becoming a serious public health concern. Global deaths and disability-adjusted life years attributable to LBMD increased from 207,367 and 8,588,936 in 1990 to 437,884 and 16,647 466 in 2019, with a raise of 111.16% and 93.82%, respectively (5). Furthermore, osteoporosis can increase hospitalization rates due to associated secondary complications, There are more than 8.9 million osteoporotic fractures worldwide. In other words, an osteoporotic fracture occurs every three seconds (6). Osteoporosis has become a notable public health problem and markedly increased healthcare expenditure. Studying the pathological mechanism of osteoporosis may facilitate decreased expenditure and improved quality of life of older adults.

Due to the large amount of energy required by the internal bone environment to maintain bone homeostasis (7), research on energy metabolism processes in the osteoporosis microenvironment has increased (8,9). Energy metabolism includes pathways that produce energy in the form of adenosine triphosphate (ATP) from nutrients, such as carbohydrate, fat and protein. Both anabolic and catabolic pathways are catalyzed by enzymes that require cofactors and ATP for activation (10). In addition to enzymatic activity, proteins [combinations of >20 amino acids (AAs)] serve as functional molecules (such as cell components, receptors, cytoskeleton and growth factors) in cells, extracellular matrices and circulatory systems. AAs needed to produce them are derived from dietary and/or cellular protein degradation, as well as synthesis through metabolic pathways, such as glycogen production and the tricarboxylic acid (TCA) cycle (also known as the citric acid cycle). The TCA cycle is dependent on carbohydrate and fatty acid metabolic pathways that are key for bone homeostasis (11,12). AAs are basic components of collagen (the primary component of the bone matrix) (13,14) and other bone-associated proteins (such as osteocalcin and alkaline phosphatase) (15,16). Therefore, AA metabolism disorders lead to a variety of pathologies, including those affecting the bone tissue (17–19). There are nine essential AAs (EAAs): Histidine, lysine, tryptophan (Trp), phenylalanine (Phe), methionine, threonine, isoleucine (Ile), leucine (Leu) and valine (Val). These AAs, including branched-chain amino acids (BCAAs) with aliphatic side chains and branched-chain structures, have a notable impact on bone formation and degradation (20,21).

An imbalance in AA acid metabolism is a key driver of the development of osteoporosis, which can aggravate bone loss by affecting bone cell energy supplies, epigenetic modification and the immune microenvironment. The balance of bone remodeling is restored by intervening in specific AA metabolic pathways, thereby decreasing bone loss (22–24). Although previous studies have revealed the regulatory role of energy metabolism (such as glucose and fatty acid metabolism) in bone homeostasis (25,26), a knowledge gap remains regarding the effects of AA metabolism on osteoporosis. To the best of our knowledge, the specific mechanism by which AA categories (such as BC and aromatic AAs) affect bone mineral density (BMD) and quality by regulating the function of key cells in the bone microenvironment has not been systematically elucidated, and the clinical transformation potential of AA metabolism intervention strategies need to be explored. The purpose of the present review is to address the limitations of traditional single-mechanism research by integrating metabolomics, genetics and cell biology evidence, combined with Mendelian randomization (MR) and computer simulation technology, to summarize the differential regulatory networks of AA categories (BCAA, aromatic AAs and glutamine) in osteoblasts, osteoclasts and bone marrow mesenchymal stem cells (BMSCs), systematically analyze the multiple mechanisms of AA metabolism in osteoporosis and ways in which it affects bone homeostasis by regulating the bone microenvironment and evaluate the potential therapeutic value of metabolic intervention. The present review aimed to provide understanding of the metabolic heterogeneity of the bone microenvironment and a scientific foundation for the development of novel metabolic therapies.

## 2. AA metabolism and osteoporosis

BCAAs, such as Val, Leu and Ile, serve important roles in bone health. Cui *et al* (27) conducted a two-sample MR analysis using inverse variance weighting, the MR-Egger method, the weighted median and the MR robust adjustment profile score to estimate the association between eight AA levels and BMD values. Hereditary increases in Ile and Val levels are positively associated with total body BMD (27). These findings highlight the key role of BCAAs in the development of osteoporosis and provide evidence that the intake of certain BCAAs can prevent and treat osteoporosis. Liao *et al* (28) confirmed that increased BCAA intake is associated with a lower risk of decreased physical function, indicating that higher dietary BCAA intake may be beneficial for physical function in the older adult population.

However, in a previous study, older adult individuals were divided into two groups. One group received 0.8 citrulline and 1.6 g Leu twice daily for 20 weeks, and the other group received a placebo. Both groups exercised independently. The results revealed no difference in BMD or bone area between the groups (29). The lack of a significant positive effect of citrulline and Leu combined with exercise may have been due to the small sample size, insufficient dose, the short intervention time, limited AA intake in the control group, limited increase in plasma Leu levels, a low basic body mass index (BMI), insufficient dietary control or low exercise intensity. These factors may have masked the potential effects of citrulline and Leu on body composition and physical activity. In another study, Leu-rich whey protein drinks were provided to individuals aged between 60 and 90 years of age for 16 weeks (30). Although the aforementioned study revealed that protein supplementation and resistance training were beneficial for certain cardiovascular metabolic indicators (such as low-density lipoprotein levels), the overall intervention effect was limited, potentially due to insufficient time, low exercise intensity, poor protein compliance and limited statistical power (30). Therefore, further research is needed on intestinal and osteoporosis microenvironments. Future studies should optimize the intervention design to verify the effect of proteins on the metabolic health of older individuals. The intake of proteins and AAs in the daily diet should be controlled and the study time should be prolonged to observe long-term effects to evaluate the role of AAs in older individuals with low BMI.

AAs containing a benzene ring, such as Phe, tyrosine and Trp, are aromatic. Phe and Trp are EAAs for human nutrition. As Phe is structurally similar to tyrosine, it can be converted into tyrosine by hydroxylation in the liver and kidney. Aromatic AAs stimulate anabolic metabolic pathways associated with bone remodeling under physiological conditions and have a positive effect on bone mass maintenance *in vivo* (20,31). Studies have revealed that in the environment of osteoporosis, Trp, an EAA for BMD, is damaged, and its metabolic pathway may be abnormal, which affects bone health (32,33). Kim *et al* (34) and Apalset *et al* (35) revealed that levels of kynurenine markedly increase in individuals with reduced hip bone mass. Kynurenine is a key intermediate of Trp metabolism in humans. Apalset *et al* (35) revealed a negative association between the kynurenine/Trp ratio and hip BMD. Ling *et al* (36) used targeted metabolomic techniques to analyze fecal samples from patients with osteoporosis and revealed that lumbar spine tyrosine and femoral neck Trp levels are higher compared with the normal group. However, by contrast with the results reported by Apalset *et al* (35), there were differences in the levels of Trp metabolism (35), which may be due to the different metabolites of bone loss analyzed and sample sources. Therefore, further research is needed to explore the association between different tissue, sample biomarkers and the pathogenesis of osteoporosis, especially the association between osteoporosis epidemic sites and characteristic metabolites.

Glycine is a common metabolite associated with BMD. Miyamoto *et al* (37) reported that the levels of serum glycine are considerably increased in patients with osteoporosis. Zhang *et al* (38) analyzed plasma samples using liquid chromatography-tandem mass spectrometry and revealed that increased levels of glycine are associated with decreased BMD in the femoral neck and lumbar vertebrae. The aforementioned studies indicated that glycine concentration is positively associated with the occurrence of osteoporosis. In addition, Eriksson *et al* (39) reported that in 965 elderly male patients (aged 69–81 years), femoral neck fractures are associated with the level of glycine circulation. A previous study revealed that the plasma level of glycine (a non-EAA) was negatively associated with BMD in male patients with idiopathic osteoporosis, whereas the levels of EAAs are normal. However, Jennings *et al* (40) and Kim *et al* (41) revealed that oral glycine has a protective effect on bones in female patients. Li *et al* (42) hypothesized that since glycine has a high affinity for estrogen receptor α, it may stimulate bone formation via specific estrogen-receptor-associated signaling pathways.

Future research should focus on exploring the deeper reasons for sex differences. Wang *et al* (43) used mass spectrometry (MS) technology to analyze serum samples from patients with osteoporosis and revealed that arginine, glutamine, histidine and serine levels in males, and glycine and hydroxyproline (t4-OH-Pro) levels in postmenopausal patients are associated with BMD. Glutamine can regulate bone metabolism by osteoclasts and trigger the bone resorption mediated by glutamate receptors on osteoblasts via conversion to glutamate (44). Sex differences in amino acid metabolism may be due to heterogeneity in hormone regulation, metabolic pathways and physiological characteristics (45). In the future, it is necessary to promote accurate diagnosis and develop sex-specific intervention strategies for osteoporosis through multicenter large-scale research, mechanism exploration and multi-omics integration.

In addition, studies (36,46) have demonstrated that BCAAs, aromatic AAs, and glycine affect osteoporosis through the intestinal microecology. Intestinal microorganisms metabolize BCAAs into short-chain fatty acids (SCFAs). SCFAs inhibit inflammation by regulating immune cells [such as regulatory T (Treg) cells] (47), and the inhibition of inflammation can inhibit the activation of osteoclasts, thereby delaying the development of osteoporosis. In addition, SCFAs promote osteoblast differentiation by activating the free fatty acid receptor 2 (11), thereby inhibiting the progression of osteoporosis. Ling *et al* (36) used ultra-high-performance liquid chromatography-MS/MS to analyze targeted metabolomics in the feces (15 categories) and serum (12 categories) of 971 participants. The results demonstrated that Val, Leu and Ile degradation were associated with the identified microbial biomarkers and osteoporosis. The aforementioned large-scale population study provides evidence that intestinal dysbacteriosis and fecal and serum metabolites are associated with osteoporosis. This is consistent with osteoporosis-related metabolomic studies by Palacios-González *et al* (48,49). Aromatic AAs can be metabolized by the intestinal flora into bioactive molecules such as SCFAs, indole derivatives and phenolic compounds, which affect bone metabolism. Trp is metabolized to indole derivatives (such as indole-3-propionic acid) by the intestinal flora, activating the aromatic hydrocarbon receptor (AhR) (50). AhR directly promotes the transcription of nuclear factor of activated T cell cytoplasmic 1 (NFATc1), the core transcription factor of osteoclast differentiation (51), which promotes osteoclast formation, and thus, promotes the development of osteoporosis. Glycine promotes the growth of beneficial bacteria (such as lactic acid bacteria and *Bifidobacteria*), improves the intestinal microecology (52), inhibits osteoclast activity and decreases osteoporosis. Gao (53) revealed that treatment of diabetic mice with *Lactobacillus* by gavage markedly increases the abundance of probiotics, alleviates insulin resistance and delays osteoporosis. Amar *et al* (54) demonstrated that, following intragastric administration of *Bifidobacterium* and *Lactobacillus*, diabetic mice exhibit significantly decreased levels of *Enterobacteriaceae* and protein levels of TNF-α, IL-1β, plasminogen activator inhibitor-1, IL-6 and IFN-γ and improved insulin sensitivity, thereby promoting bone formation and repair (55).

Generally, an imbalance in AA metabolism is a notable characteristic in patients with osteoporosis (56). Osteoporotic cells are primarily composed of osteoblasts, osteoclasts and bone-marrow-derived stem cells. Therefore, it is important to study the metabolism of AAs in the osteoporotic microenvironment.

## 3. AA metabolism in osteoblasts

Osteocytes are the main regulators of bone homeostasis, which is achieved by the regulation of bone formation by osteoblasts and bone absorption by osteoclasts (57). Recent studies have revealed that a lack of EAAs can lead to the phosphorylation of MAPK, which leads to cell cycle arrest, thereby inhibiting cell proliferation and osteogenic differentiation (58–60). In addition, the lack of EAAs induces reactive oxygen species-mediated DNA damage and apoptosis (60), confirming the role of EAAs in osteoblasts.

**Figure 1.**
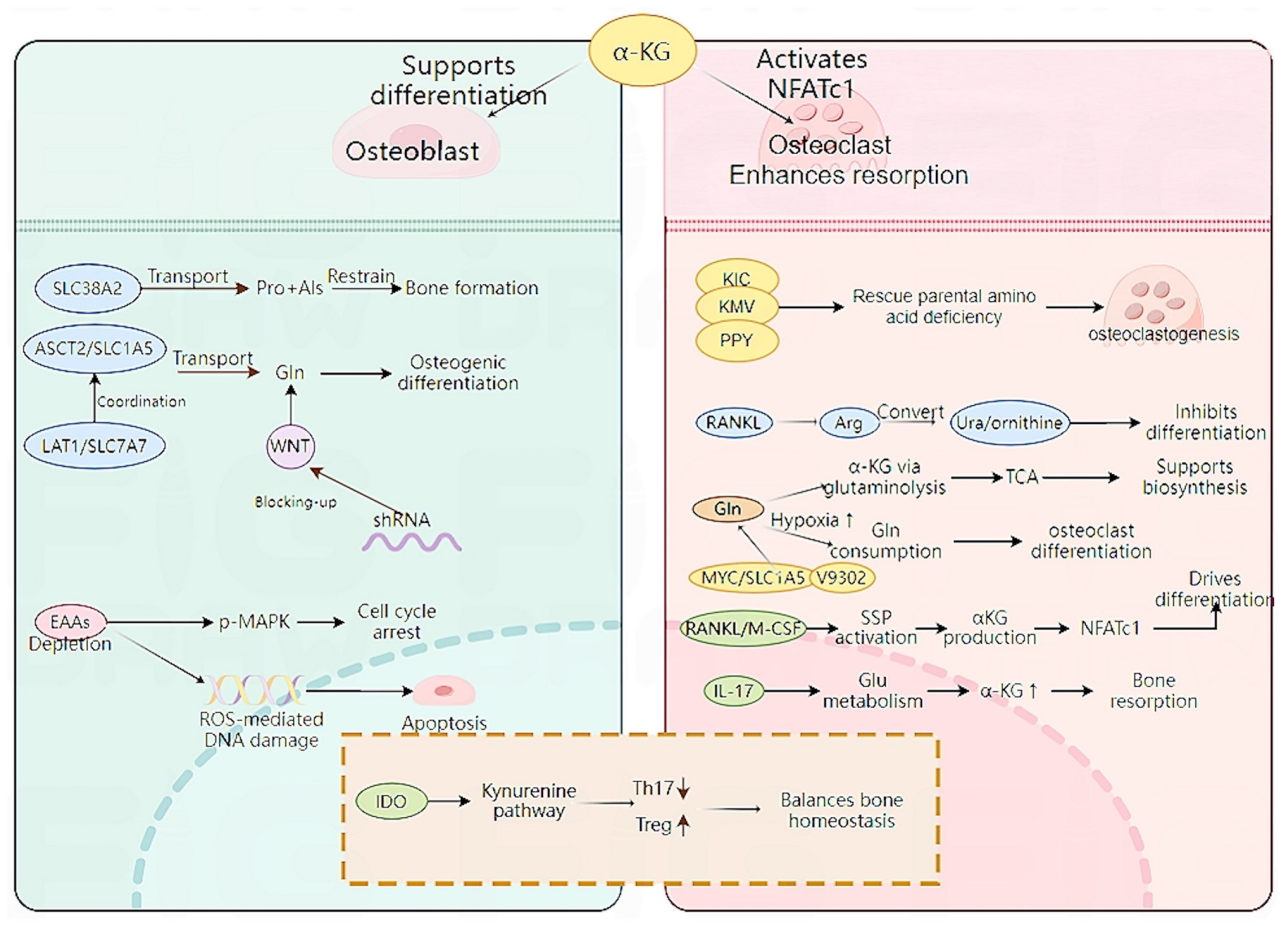
EAAs are crucial for osteoblast function; their deficiency activates MAPK signaling, causing cell cycle arrest, ROS accumulation, DNA damage and apoptosis. AA transporters also serve key roles. SLC38A2 mediates proline and alanine uptake, and its deletion decreases bone mass in mice. SLC1A5 supplies glutamine and asparagine, key for protein synthesis and osteoblast differentiation. SLC1A5 and SLC7A7 (γ^+^-LAT1) coordinate glutamine uptake in response to Wnt signaling. Knockdown of these transporters impairs glutamine uptake and blocks Wnt-induced osteogenesis. Osteoclastogenesis depends on dynamic AA metabolism. Key EAA-derived metabolites (from Leu, Ile and Phe) promote differentiation under AA deficiency. Arginine is key for RANKL-induced osteoclast formation; its depletion impairs differentiation independently of mTORC1 and can be rescued by citrulline or argininosuccinate. Glutamine supports biosynthesis and energy production via α-ketoglutarate (αKG), activates NFATc1 epigenetically through the serine synthesis pathway, and is required in late differentiation via SLC1A5 and GLS. Hypoxia increases glutamine demand, and MYC inhibition suppresses this pathway. IL-17-αKG signaling contributes to bone loss and is blocked by SLC1A5 inhibitor V9302. Tryptophan catabolism via IDO regulates Th17/Treg balance, indirectly affecting osteoclast activity. BMSC, bone marrow mesenchymal stem cell; EAA, essential amino acid; ROS, reactive oxygen species KIC, α-Ketoisocaproate; KMV, α-Keto-β-methylvalerate; PPY, Phenylpyruvate; αKG, α-Ketoglutarate; ROS, reactive Oxygen Species; M-CSF, Macrophage Colony-Stimulating Factor; TCA, Tricarboxylic Acid Cycle; SSP, Serine Synthesis Pathway; IDO, Indoleamine 2,3-Dioxygenase; Th17, T Helper 17 Cells; Treg, Regulatory T Cells; TGF-β, Transforming Growth Factor-β; hMSC, human Mesenchymal Stem Cell; GLS, Glutaminase; GDH, Glutamate Dehydrogenase; AhR, aryl Hydrocarbon Receptor; shRNAs, short hairpin RNAs; SLC38A2, Solute Carrier Family 38 Member 2; NFATc1, Nuclear Factor of Activated T Cells, Cytoplasmic 1.

In addition to the effect of EAAs on osteoblasts, the neutral AA solute carrier family 38 member 2 (SLC38A2) provides proline and alanine to osteoblasts. In mice, ablation of SLC38A2 results in a decreased bone mass, highlighting the role of SLC38A2-mediated proline and alanine intake for postpartum bone formation and bone homeostasis (61). In addition, genetic and metabolomics studies have demonstrated that the AA transporter cysteine transporter 2 (SLC1A5, encoded by Slc1a5) provide glutamine and asparagine, thereby regulating protein synthesis and osteoblast differentiation (62,63). AA transporters, system γ(+)-L transporter γ(+)-L transporter 1 [γ (+)-LAT1] and SLC1A5 (encoded by SlC7A7 and SlC1A5, respectively), are the primary transporters of glutamine in response to Wnt and SLC1A5 mediates the majority of glutamine intake (64). Using short hairpin RNAs targeting SlC7A7 or SlC1A5 decreases Wnt-induced glutamine intake, thereby preventing osteoblast differentiation (64). The mechanism of AA metabolism in osteoblasts is shown in Fig. 1. In summary, the aforementioned studies demonstrated the key role of EAAs and associated transport genes in osteoblasts and revealed the potential for targeting AA metabolism to interfere with bone formation and resorption (Table I).

## 4. AA metabolism in osteoclasts

During osteoclast development, metabolic pathways change, especially AA metabolism, and this serves a key role in the regulation of osteoclast formation. EAAs α-ketoisocaproate, α-keto-β-methylvalerate and phenylpyruvate, the intermediates of Leu, Ile and Phe metabolism, respectively (4), serve a key role in the formation of osteoclasts. These amino acids can effectively alleviate the inhibition of osteoclast formation because of a lack of parental AAs (65). Osteoclasts contain a large number of intracellular proteins involved in lysine decomposition, which stimulate the biosynthesis of tyrosine, Phe and Trp (66). Osteoclasts are rich in intracellular proteins involved in lysine degradation, which activates the biosynthesis of tyrosine, Phe and Trp (67). Recent studies have revealed that receptor activator of nuclear factor-κB ligand (RANKL)-induced osteoclast formation is primarily dependent on the presence of extracellular arginine (68,69). RANKL-induced proteins are antagonized by recombinant arginase 1, which metabolizes arginine to urea and ornithine. Excessive arginine intake can restore osteoclastogenesis by supplementing arginine-succinic acid and citrulline, but direct supplementation of TCA intermediates, such as α-ketoglutarate (αKG), has no effect (65). The effect of arginine deficiency on osteoclast formation is not affected by mTORC1 activity or the inhibition of overall transcription and translation (67). In patients with RA and pre-RA, L-arginine effectively inhibits the progression of arthritis and bone loss, and can directly block TNFα-induced osteoclastogenesis in mice and humans (8).

Osteoclast differentiation is synergistically affected by macrophage colony-stimulating factor and RANKL, which activate signaling pathways and interact with each other to regulate the expression and function of the key transcriptional regulator NFATc1. Early in the process of osteoclast formation, αKG produced by RANKL-induced serine synthesis pathway activation regulates the expression of Nfatc1 by epigenetics, thereby promoting osteoclast differentiation (70). In addition to facilitating protein synthesis, glutamine is an important energy source and a carbon and nitrogen donor for the synthesis of AAs, nucleotides, glutathione and aminohexose. It is converted to αKG via glutamine decomposition, enters the TCA cycle, and is converted to citric acid (71,72). During osteoclast differentiation, the expression of Na^+^-dependent glutamine neutral AA transporter B increases, which serves a key role in the later stage of differentiation (73). In addition, Tsumura *et al* (74) revealed that a hypoxic environment can stimulate osteoclasts to consume glutamine. The inhibition of MYC can effectively prevent osteoclast differentiation and function and inhibit the expression of SLC1A5 and glutaminase (GLS). Therefore, glutamine uptake is key for osteoclast development and bone resorption (75). Although glutamine metabolism may indirectly support energy supply by providing intermediates of the TCA cycle, its primary role is to promote the synthesis of biomolecules that accelerate osteoclast differentiation and functional maturation (44). During the development of osteoporosis, osteoclasts require ATP to exert their bone resorption function. Glu and its downstream metabolite αKG are involved in the IL-17-mediated pathway *in vivo* to aggravate ovariectomy-induced bone loss, which can be inhibited by V9302 (an SLC1A5 inhibitors), thereby interfering with osteoclast differentiation and bone resorption (76).

An imbalance of osteoblast and osteoclast activity is a key factor in AA metabolism (77). Specific T cell subsets, such as regulatory (Treg) and helper T cells 17 (Th17) are involved in the imbalance between osteoblast and osteoclast activity (78). Indoleamine 2,3-dioxygenase, a rate-limiting enzyme of Trp catabolism, serves a role in the kynurenine pathway and may be a key protein in regulating the balance ratio of Th17/Treg cells (79). Its metabolites inhibit Th17 cell differentiation and promote Treg cells production, thereby affecting the balance between Th17 and Treg cells (80). In summary, amino acid metabolism is associated with osteoclasts (Fig. 1; Table I).

## 5. AA metabolism in bone marrow mesenchymal stem cells

Eagle *et al* (81) studied the role of glutamine in the proliferation of mesenchymal stem cells. Colombo *et al* (82) and Ahn *et al* (83) used advanced pulse-tracking, liquid chromatography-MS, isotope tracing technology, computational deconvolution and metabolic flux modeling techniques to confirm that glutamine is an essential substrate that specifically leads from the S phase to cell division. Glutamine can also increase the expression of cyclin D1 and D3, promote the initiation of S phase and inhibit the expression of p21, a key regulator of the G1/S cycle checkpoint (84,85). Notably, this may involve GLS, because glutamine can increase the activity of GLS and glutamate dehydrogenase in a dose-dependent manner via the mTOR/S6 and MAPK pathways, thereby promoting cell proliferation (86). However, the underlying mechanism remains unclear. In addition, glutamine not only provides precursors for DNA replication in S phase and lipid synthesis in G2 phase, but also degrades glutamine in endothelial cells, which is the primary source of TCA cycle carbon and nitrogen for some non-EAAs (87).

BMSCs are progenitor cells with potential to differentiate into bone, fat and cartilage lineages (88). Previous studies have revealed that human and mouse BMSCs consume large amounts of glutamine during differentiation (71,89). During this process, glutamine metabolism produces ATP through the TCA cycle to meet the energy needs of physiological functions (72). In addition, GLS has also been demonstrated to promote the differentiation of BMSCs into osteoblasts (90). However, when BMSCs lack GLS, there is a decrease in the total number of osteoblasts and bone formation ability (89). Yu *et al* (72) also revealed that the proliferation and colony expansion of BMSCs is dependent on the production of AA transaminase-dependent αKG, which explains the adverse effects of decreased GLS activity on the proliferation of BMSCs. Other studies have revealed that the glutamine metabolite αKG improves the osteogenic potential of BMSCs by decreasing histone methylation (91,92). These results suggest that glutamine and αKG may promote the osteogenic differentiation of BMSCs. Therefore, in-depth study on glutamine metabolism in BMSCs may provide novel strategies and methods for the treatment of bone loss.

**Figure 2.**
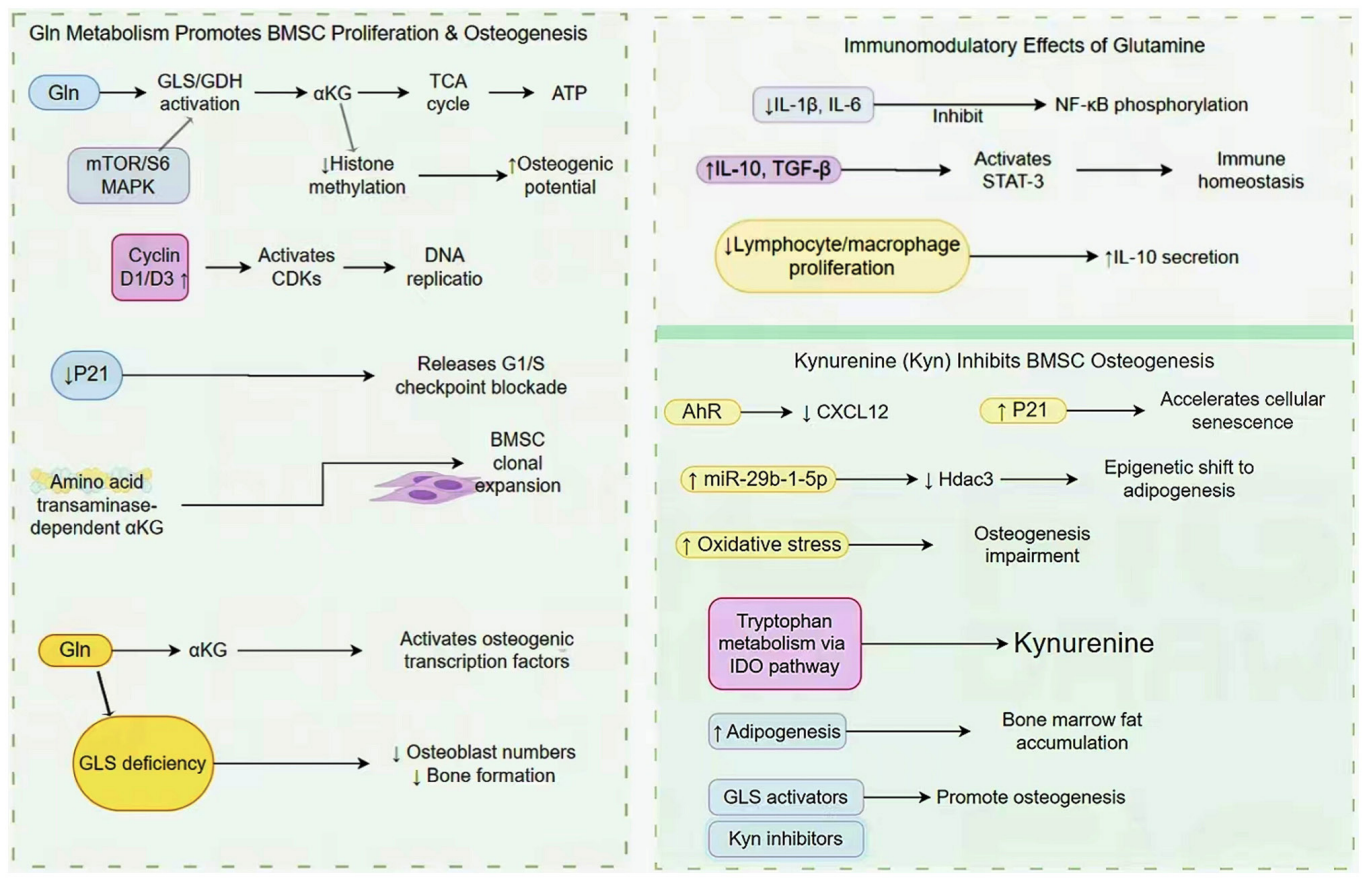
Glutamine not only promotes the proliferation and osteogenic differentiation of BMSCs via mTOR/S6 and MAPK pathways, but also improves the inflammatory microenvironment by inhibiting pro-inflammatory factors such as IL-1β or IL-6 and enhancing anti-inflammatory factors such as IL-10 or TGF-β; metabolites such as kynurenine inhibit bone formation by promoting adipogenesis and apoptosis of BMSCs, while AA metabolism disorders break the dynamic balance of osteogenesis and osteoclasts via multiple pathways such as inflammation, oxidative stress and energy imbalance, leading to bone loss. AhR, aryl Hydrocarbon Receptor; BMSC, Bone Marrow Mesenchymal Stem Cell; CDKs, cyclin-Dependent Kinases; CXCL12, C-X-C Motif Chemokine Ligand 12; GLS, Glutaminase; GDH, Glutamate Dehydrogenase; αKG, Alpha-Ketoglutarate; Hdac3, Histone Deacetylase 3; IDO, Indoleamine 2,3-Dioxygenase; Kyn, Kynurenine; miR-29b-1-5p, microRNA-29b-1-5p; TCA, Tricarboxylic Acid Cycle.

In BMSCs, the glutamine concentration directly affects immune characteristics. High concentrations of glutamine effectively inhibit inflammation, potentially by decreasing activity of pro-inflammatory cytokines, such as IL-1β and IL-6, and increasing the expression levels of the anti-inflammatory cytokines IL-10 and transforming growth factor-β (93). The mechanism mainly involves the regulation of cytokines by decreasing the levels of phosphorylated NF-κB and signal transduction and activator 3 (STAT-3) (94). In particular, IL-10, an important anti-inflammatory cytokine that can hinder the activity of NF-κB and regulate cytokine production (95). Studies (96–98) have shown that glutamine concentration has a regulatory effect on IL-10 expression. Adequate glutamine supply upregulates IL-10 expression and enhances its anti-inflammatory function; glutamine deficiency leads to decreased IL-10 expression and impairs its inhibitory effect on the NF-κB signaling pathway, thereby exacerbating the inflammatory response (99,100). IL-10 can also activate STAT-3, thereby decreasing the levels of pro-inflammatory cytokines (101,102). In addition, proliferation of lymphocytes and macrophages in BMSCs decreases following glutamine exposure and the secretion of IL-10 increases (103). At 4 weeks following the intraperitoneal injection of kynurenine (10 mg/kg) in adult mice, the osteogenic differentiation ability of BMSCs decreases considerably, accompanied by the deterioration of osteoblast bioenergetics and productivity (104). *In vitro* studies have revealed that human pluripotent stem cells stimulated by kynurenine have altered microRNA (miRNA or miR) expression levels, resulting in increased oxidative stress and the inhibition of osteogenic differentiation (105,106). The C-X-C motif chemokine ligand 12 (CXCL12) protein, a necessary mediator of the osteogenic differentiation of BMSCs, is also notably affected by kynurenine. Kynurenine decreases its expression through the AhR signaling pathway (107). Kynurenine also increases the levels of P21 and cell death (108). In addition, it causes BMSCs to develop mainly in the direction of adipogenesis by increasing the expression level of miR-29b-1-5p and downregulating the levels of histone deacetylase-3 (Hdac3) and CXCL12, creating a toxic bone marrow environment, thus exacerbating bone loss and increasing the fracture risk (109). The mechanism of AA metabolism in BMSCs is shown in Fig. 2. In summary, kynurenine regulates the levels of miRNAs, proteins and activity of metabolic pathways to affect the osteogenic differentiation ability of BMSCs (Table II).

## 6. Summary and prospects

Osteoporosis is a metabolic disease characterized by an imbalance in bone remodeling. Its pathology is caused by dysfunction of osteoblasts, osteoclasts and BMSCs. Recent studies have demonstrated that AA metabolism serves a key role in the occurrence and development of osteoporosis by regulating energy supply, signal transduction, epigenetic modification and immune homeostasis of the bone microenvironment (20,44,110). The present review summarized the differential regulatory networks of types of AA, such as BCAAs, aromatic AAs and glutamine, in bone homeostasis and the mechanisms underlying their effects on osteoporosis by interfering with the functions of osteoblasts, osteoclasts and bone marrow mesenchymal stem cells, as well as application prospects of research methods based on metabolomics and genetics for promoting the development of precise treatment.

Although several studies have revealed the role of AA metabolism in osteoporosis, they have limitations (9,20,56,111). First, the specific regulatory mechanisms of AAs in bone metabolism have not yet been fully elucidated. For example, to the best of our knowledge, a systematic analysis of the roles of BCAAs, aromatic AAs and glutamine in different bone cells (osteoblasts, osteoclasts and BMSCs) is lacking (112). In addition, the specific regulatory network of AA metabolism in the bone microenvironment is complex and is affected by numerous factors, including gene expression, signaling pathways, the immune microenvironment and energy metabolism (21). However, the majority of studies are limited to exploring a single mechanism and do not reveal the overall regulatory model (20,56). Although observational and genetic association studies have revealed an association between AA metabolism and the occurrence and development of osteoporosis, randomized controlled trials based on metabolic interventions are limited (9,27). For example, there is a lack of long-term intervention data demonstrating that BCAA supplementation can improve BMD and decrease fracture risk (113). In addition, certain studies have problems such as a small sample size, a short intervention time and insufficient dietary control, resulting in inconsistent results and affecting its clinical transformation value (114,115). In addition, individual differences such as sex, age, genetic background and lifestyle may lead to differences in AA metabolism patterns. For example, female patients are more affected by changes in estrogen levels, whereas male patients may be more affected by androgens and other metabolic pathways (116). Therefore, future studies should consider individual factors to improve the applicability of the results.

In future, the mechanisms of AA metabolism in osteoporosis should be elucidated. In addition, new animal models of osteoporosis should be developed using specific metabolic markers. This may provide a novel perspective for the direct visual analysis of bone cell metabolic changes to develop drug, prevention and treatment concepts. The effects of AA metabolism on the energy supply and epigenetic modification of bone cells should be explored. In clinical practice, a larger-scale, long-term follow-up clinical intervention trial should be designed to evaluate the effects of AA supplementation on BMD, fracture risk and quality of life in patients with osteoporosis and optimize the clinical transformation of AA metabolism intervention strategies. In addition, the application of individualized medicine should be strengthened by combining genomic and metabolomic data to explore the differences in the responses of individuals to AA metabolism interventions. Artificial intelligence and machine-learning techniques can be used in combination with big data analysis to establish predictive models to assess the risk of individual AA metabolism patterns in osteoporosis and provide personalized nutrition intervention recommendations. In recent years, more evidence has revealed that the gut microbiota serve a key role in the development of osteoporosis (117,118). It is of clinical value to explore the interactions between AA metabolism and intestinal microecology. Specifically, RNA sequencing, metagenomic and metabolomic analyses should be used to study the composition of the intestinal flora and its association with AA metabolism in different groups of patients with osteoporosis. In addition, the effects of probiotics, prebiotics and specific AA supplementation on BMD should be evaluated to explore the regulation of the intestinal microecology as a novel strategy for the treatment of osteoporosis.

## Figures and Tables

**Table I.** Application of amino acid metabolism in osteoblasts/osteoclasts.

First author/s, year	Targeted bone cells	Amino acid intervention or involvement	Mechanism	Results	(Refs.)
Li *et al*, 2023	Osteoblast	Lack of essential amino acids	Leads to phosphorylation of MAPK signaling pathway; induces reactive oxygen species	Induces cell cycle arrest, inhibits cell proliferation and osteogenic differentiation; mediates DNA damage and apoptosis	(59)
Shen *et al*, 2022	Osteoblast	Proline and alanine	SLC38A2 gene ablation	Bone mass loss in mice	(62)
Sharma *et al*, 2021; Jiménez *et al*, 2022	Osteoblast	Glutamine and asparagine	SLC1A5 provides glutamine and asparagine for osteoblast differentiation	Regulates protein synthesis and osteoblast differentiation	(63,64)
Shen *et al*, 2021	Osteoblast	Glutamine	Short hairpin RNA targeting SlC7A7 or SlC1A5 decreases WNT-induced glutamine intake	Prevents osteoblast differentiation	(65)
Nie *et al*, 2018;	Osteoclast	Leucine	Α-ketoisocaproate	Restores the inhibitory	(4,66)
Lademann *et al*,	Osteoclast	Isoleucine	Ketoisoleucine	effect of lack of parental	
2020	Osteoclast	Phenylalanine	p-phenylpyruvic acid	amino acids on osteoclastogenesis	
Nie *et al*, 2018; Onuora *et al*, 2023 Shen *et al*, 2024	Osteoclast	Arginine	Recombinant arginase 1 can antagonize RANKL-induced protein	Inhibition of osteoclast formation induced by receptor activator of nuclear factor-κB ligand (RANKL)	(66,69,70)
Stegen *et al*	Osteoclast	Glutamine	Glutamine is decomposed into	Promote osteoclast	(71,72,73)
2024; Zhou *et al*,			differentiation αKG,	which enters the	
2019; Yu *et al*,			tricarboxylic acid cycle and is		
2019			converted into citric acid.		
Hu *et al*, 2024	Osteoclast	Glutamine	Promote the synthesis of biological macromolecules	Accelerating the differentiation and functional maturation of osteoclasts	(44)
Peng *et al*, 2024	Osteoclast	Glutamic acid	Downstream metabolite αKG	IL-17 aggravates bone loss.	(77)
Zhang *et al*, 2020;			Indoleamine 2,3-dioxygenase	Metabolites inhibit	
Mellor *et al*, 2004	Osteoclast	Kynurenine	may be a key protein regulating the balance ratio of Th17/Treg cells	generation of Treg cells, affecting the the differentiation of Th17 cells and promote the balance of Th17 / Treg.	(79,80)

αKG, α-ketoglutarate; SLC38A2, solute Carrier Family 38 Member 2; p-, phosphorylated; shRNA, Short Hairpin RNA; ROS, Reactive Oxygen Species; RANKL, Receptor Activator of Nuclear Factor-κB Ligand; RecArg1, Recombinant Arginase 1; TCA, Tricarboxylic Acid Cycle; Th17, T Helper 17 Cells; Treg, Regulatory T Cells; IDO, Indoleamine 2,3-Dioxygenase.

**Table II.** Application of amino acid metabolism in BMSCs.

First author/s, year	Amino acid and metabolite involvement	Mechanism	Outcome	(Refs.)
Malakar *et al*, 2023;	Glutamine	Enhanced expression of	Accelerate BMSC	(85,86)
Minchenko *et al*, 2011		cyclin D1 and D3	proliferation	
Yuan *et al*, 2015	Glutamine	GLS and glutamate dehydrogenase activity increased in a dose-dependent manner through mTOR/S6 and MAPK pathways	Accelerate BMSC proliferation	(87)
Skerry *et al*, 2008;	Glutamine	GLS	Promotes the differentiation	(90,91)
Ning *et al*, 2022			of BMSCs into osteoblasts. When BMSCs lack GLS, the number of osteoblasts and bone formation decreases	
Wang *et al*, 2020;	Glutamine	αKG decreases histone	Improves osteogenic	(92,93)
fan *et al*, 2022		methylation	potential of BMSCs	
Qian *et al*, 2017;	Glutamine	Decreases expression of	Strong osteogenic	(94,95,97–101)
Ganesan *et al*, 2017;		pro-inflammatory	differentiation ability and	
Santos *et al*, 2016;		cytokines such as IL-1β	immune regulation	
da Silva Lima *et al*, 2013;		and IL-6 and increases	function of BMSCs	
sun *et al*, 2019;		anti-inflammatory		
Levy *et al*, 2002;		cytokines such as IL-10,		
Dos Santos *et al*, 2017		STAT-3 and TGF-β; IL-10 activates STAT-3, decreasing the levels of pro-inflammatory cytokines		
Wang *et al*, 2020	Glutamine	The glutamine metabolite αKG reduces histone methylation	Improves the osteogenic potential of BMSCs	(92)
Dalton *et al*, 2020;	Kynurenine	Human pluripotent stem	Increased oxidative stress	(103,104)
Sas *et al*, 2018		cells stimulated by kynurenine exhibit altered expression level of miR	and inhibited osteogenic differentiation	
Elmansi *et al*, 2020	Kynurenine	Kynurenine decreases CXCL12 expression through the AhR signaling pathway	Decreases CXCL12 protein levels and osteogenic differentiation	(105)
Anaya *et al*, 2020	Kynurenine	Upregulation of miR-29b-1-5p and downregulation of Hdac3 and CXCL12	Promotes development of BMSCs in the direction of adipogenesis, causing toxic bone marrow environment, exacerbating bone loss and increasing the risk of fracture	(107)

GLS, glutaminase; GDH, glutamate dehydrogenase; αKG, α-ketoglutarate; BMSC, bone marrow mesenchymal stem cell; CXCL12, C-X-C motif chemokine ligand 12; AhR, aryl hydrocarbon receptor; miR microRNA; Hdac, histone deacetylase-3.

## Data Availability

Not applicable.

## References

[1] Weaver CM, Gordon CM, Janz KF, Kalkwarf HJ, Lappe JM, Lewis R, O'Karma M, Wallace TC, Zemel BS (2016). The National Osteoporosis Foundation's position statement on peak bone mass development and lifestyle factors: A systematic review and implementation recommendations. Osteoporos Int.

[2] Rozenberg S, Bruyère O, Bergmann P, Cavalier E, Gielen E, Goemaere S, Kaufman JM, Lapauw B, Laurent MR, De Schepper J, Body JJ (2020). How to manage osteoporosis before the age of 50. Maturitas.

[3] Wu Y, Xie L, Wang M, Xiong Q, Guo Y, Liang Y, Li J, Sheng R, Deng P, Wang Y (2018). Mettl3-mediated mA RNA methylation regulates the fate of bone marrow mesenchymal stem cells and osteoporosis. Nat Commun.

[4] Lademann F, Tsourdi E, Hofbauer LC, Rauner M (2020). Thyroid hormone actions and bone remodeling-the role of the wnt signaling pathway. Exp Clin Endocrinol Diabetes.

[5] Shen Y, Huang X, Wu J, Lin X, Zhou X, Zhu Z, Pan X, Xu J, Qiao J, Zhang T (2022). The Global Burden of osteoporosis, low bone mass, and its related fracture in 204 countries and territories, 1990–2019. Front Endocrinol (Lausanne).

[6] Johnston CB, Dagar M (2020). Osteoporosis in older adults. Med Clin North Am.

[7] Confavreux CB, Levine RL, Karsenty G (2009). A paradigm of integrative physiology, the crosstalk between bone and energy metabolisms. Mol Cell Endocrinol.

[8] Cao S, Li Y, Song R, Meng X, Fuchs M, Liang C, Kachler K, Meng X, Wen J, Schlötzer-Schrehardt U (2024). L-arginine metabolism inhibits arthritis and inflammatory bone loss. Ann Rheum Dis.

[9] Panahi N, Fahimfar N, Roshani S, Arjmand B, Gharibzadeh S, Shafiee G, Migliavacca E, Breuille D, Feige JN, Grzywinski Y (2022). Association of amino acid metabolites with osteoporosis, a metabolomic approach: Bushehr elderly health program. Metabolomics.

[10] Wilson MP, Plecko B, Mills PB, Clayton PT (2019). Disorders affecting vitamin B6 metabolism. J Inherit Metab Dis.

[11] Montalvany-Antonucci CC, Duffles LF, de Arruda JAA, Zicker MC, de Oliveira S, Macari S, Garlet GP, Madeira MFM, Fukada SY, Andrade I (2019). Short-chain fatty acids and FFAR2 as suppressors of bone resorption. Bone.

[12] Lucas S, Omata Y, Hofmann J, Böttcher M, Iljazovic A, Sarter K, Albrecht O, Schulz O, Krishnacoumar B, Krönke G (2018). Short-chain fatty acids regulate systemic bone mass and protect from pathological bone loss. Nat Commun.

[13] Prockop DJ, Kivirikko KI (1995). Collagens: Molecular biology, diseases, and potentials for therapy. Annu Rev Biochem.

[14] Shoulders MD, Raines RT (2009). Collagen structure and stability. Annu Rev Biochem.

[15] Whyte MP (2010). Physiological role of alkaline phosphatase explored in hypophosphatasia. Ann N Y Acad Sci.

[16] Heaney RP, Layman DK (2008). Amount and type of protein influences bone health. Am J Clin Nutr.

[17] Ding KH, Cain M, Davis M, Bergson C, McGee-Lawrence M, Perkins C, Hardigan T, Shi X, Zhong Q, Xu J (2018). Amino acids as signaling molecules modulating bone turnover. Bone.

[18] Long F (2018). Energy metabolism and bone. Bone.

[19] Dirckx N, Moorer MC, Clemens TL, Riddle RC (2019). The role of osteoblasts in energy homeostasis. Nat Rev Endocrinol.

[20] Lv Z, Shi W, Zhang Q (2022). Role of essential amino acids in age-induced bone loss. Int J Mol Sci.

[21] Devignes CS, Carmeliet G, Stegen S (2022). Amino acid metabolism in skeletal cells. Bone Rep.

[22] Xu F, Li W, Yang X, Na L, Chen L, Liu G (2021). The roles of epigenetics regulation in bone metabolism and osteoporosis. Front Cell Dev Biol.

[23] Chen X, Huang X, Zhang X, Chen Z (2025). Metabolism-epigenetic interaction-based bone and dental regeneration: From impacts and mechanisms to treatment potential. Bone.

[24] Yang L, Chu Z, Liu M, Zou Q, Li J, Liu Q, Wang Y, Wang T, Xiang J, Wang B (2023). Amino acid metabolism in immune cells: Essential regulators of the effector functions, and promising opportunities to enhance cancer immunotherapy. J Hematol Oncol.

[25] Karner CM, Long F (2018). Glucose metabolism in bone. Bone.

[26] Alekos NS, Moorer MC, Riddle RC (2020). Dual effects of lipid metabolism on osteoblast function. Front Endocrinol (Lausanne).

[27] Cui Z, Feng H, He B, He J, Tian Y (2021). Relationship between serum amino acid levels and bone mineral density: A mendelian randomization study. Front Endocrinol (Lausanne).

[28] Liao M, Mu Y, Su X, Zheng L, Zhang S, Chen H, Xu S, Ma J, Ouyang R, Li W (2022). Association between Branched-Chain Amino Acid Intake and Physical Function among Chinese Community-Dwelling Elderly Residents. Nutrients.

[29] Kim M, Isoda H, Okura T (2021). Effect of Citrulline and leucine intake with exercises on body composition, physical activity, and amino acid concentration in older women: A Randomized double-blind placebo-controlled study. Foods.

[30] Kirk B, Mooney K, Vogrin S, Jackson M, Duque G, Khaiyat O, Amirabdollahian F (2021). Leucine-enriched whey protein supplementation, resistance-based exercise, and cardiometabolic health in older adults: A randomized controlled trial. J Cachexia Sarcopenia Muscle.

[31] Refaey ME, Zhong Q, Ding KH, Shi XM, Xu J, Bollag WB, Hill WD, Chutkan N, Robbins R, Nadeau H (2014). Impact of dietary aromatic amino acids on osteoclastic activity. Calcif Tissue Int.

[32] Michalowska M, Znorko B, Kaminski T, Oksztulska-Kolanek E, Pawlak D (2015). New insights into tryptophan and its metabolites in the regulation of bone metabolism. J Physiol Pharmacol.

[33] Akinsuyi OS, Roesch LFW (2023). Meta-analysis reveals compositional and functional microbial changes associated with osteoporosis. Microbiol Spectr.

[34] Kim BJ, Hamrick MW, Yoo HJ, Lee SH, Kim SJ, Koh JM, Isales CM (2019). The detrimental effects of kynurenine, a tryptophan metabolite, on human bone metabolism. J Clin Endocrinol Metab.

[35] Apalset EM, Gjesdal CG, Ueland PM, Midttun Ø, Ulvik A, Eide GE, Meyer K, Tell GS (2014). Interferon (IFN)-γ-mediated inflammation and the kynurenine pathway in relation to bone mineral density: The Hordaland health study. Clin Exp Immunol.

[36] Ling CW, Miao Z, Xiao ML, Zhou H, Jiang Z, Fu Y, Xiong F, Zuo LS, Liu YP, Wu YY (2021). The association of gut microbiota with osteoporosis is mediated by amino acid metabolism: Multiomics in a large cohort. J Clin Endocrinol Metab.

[37] Miyamoto K, Hirayama A, Sato Y, Ikeda S, Maruyama M, Soga T, Tomita M, Nakamura M, Matsumoto M, Yoshimura N, Miyamoto T (2021). A metabolomic profile predictive of new osteoporosis or sarcopenia development. Metabolites.

[38] Zhang X, Xu H, Li GH, Long MT, Cheung CL, Vasan RS, Hsu YH, Kiel DP, Liu CT (2021). Metabolomics insights into osteoporosis through association with bone mineral density. J Bone Miner Res.

[39] Eriksson AL, Friedrich N, Karlsson MK, Ljunggren Ö, Lorentzon M, Nethander M, Wallaschofski H, Mellström D, Ohlsson C (2021). Serum glycine levels are associated with cortical bone properties and fracture risk in men. J Clin Endocrinol Metab.

[40] Jennings A, MacGregor A, Spector T, Cassidy A (2016). Amino acid intakes are associated with bone mineral density and prevalence of low bone mass in women: Evidence from discordant monozygotic twins. J Bone Miner Res.

[41] Kim MH, Kim HM, Jeong HJ (2016). Estrogen-like osteoprotective effects of glycine in in vitro and in vivo models of menopause. Amino Acids.

[42] Li X, Lin Q, Cui Y, Wang H, Wang P, Yang L, Ye Q, Zhang R, Zhu X (2022). Glycine acts through estrogen receptor alpha to mediate estrogen receptor signaling, stimulating osteogenesis and attenuating adipogenesis in ovariectomized rats. Mol Nutr Food Res.

[43] Wang J, Yan D, Zhao A, Hou X, Zheng X, Chen P, Bao Y, Jia W, Hu C, Zhang ZL, Jia W (2019). Discovery of potential biomarkers for osteoporosis using LC-MS/MS metabolomic methods. Osteoporos Int.

[44] Hu G, Yu Y, Ren Y, Tower RJ, Zhang GF, Karner CM (2024). Glutaminolysis provides nucleotides and amino acids to regulate osteoclast differentiation in mice. EMBO Rep.

[45] Lamont LS, McCullough AJ, Kalhan SC (2003). Gender differences in the regulation of amino acid metabolism. J Appl Physiol (1985).

[46] Zhang YW, Song PR, Wang SC, Liu H, Shi ZM, Su JC (2024). Diets intervene osteoporosis via gut-bone axis. Gut Microbes.

[47] Mann ER, Lam YK, Uhlig HH (2024). Short-chain fatty acids: Linking diet, the microbiome and immunity. Nat Rev Immunol.

[48] Palacios-González B, Ramírez-Salazar EG, Rivera-Paredez B, Quiterio M, Flores YN, Macias-Kauffer L, Moran-Ramos S, Denova-Gutiérrez E, Ibarra-González I, Vela-Amieva M (2020). A Multi-Omic Analysis for Low Bone Mineral Density in Postmenopausal Women Suggests a RELATIONSHIP between Diet, Metabolites, and Microbiota. Microorganisms.

[49] Palacios-González B, León-Reyes G, Rivera-Paredez B, Ibarra-González I, Vela-Amieva M, Flores YN, Canizales-Quinteros S, Salmerón J, Velázquez-Cruz R (2021). Serum metabolite profile associated with sex-dependent visceral adiposity index and low bone mineral density in a mexican population. Metabolites.

[50] Gao J, Xu K, Liu H, Liu G, Bai M, Peng C, Li T, Yin Y (2018). Impact of the gut microbiota on intestinal immunity mediated by tryptophan metabolism. Front Cell Infect Microbiol.

[51] Ye Q, Xi X, Fan D, Cao X, Wang Q, Wang X, Zhang M, Wang B, Tao Q, Xiao C (2022). Polycyclic aromatic hydrocarbons in bone homeostasis. Biomed Pharmacother.

[52] Wang L, Wang Z, Luo P, Bai S, Chen Y, Chen W (2023). Dietary zinc glycine supplementation improves tibia quality of meat ducks by modulating the intestinal barrier and bone resorption. Biol Trace Elem Res.

[53] Gao W (2018). Effects of lactobacillus on glucolipids metabolism and intestinal flora in type 2 diabetic mice fed with high-glucose and high-fat diet (master's thesis).

[54] Amar J, Chabo C, Waget A, Klopp P, Vachoux C, Bermúdez-Humarán LG, Smirnova N, Bergé M, Sulpice T, Lahtinen S (2011). Intestinal mucosal adherence and translocation of commensal bacteria at the early onset of type 2 diabetes: Molecular mechanisms and probiotic treatment. EMBO Mol Med.

[55] Imerb N, Thonusin C, Chattipakorn N, Chattipakorn SC (2020). Aging, obese-insulin resistance, and bone remodeling. Mech Ageing Dev.

[56] Lau KT, Krishnamoorthy S, Sing CW, Cheung CL (2023). Metabolomics of osteoporosis in humans: A systematic review. Curr Osteoporos Rep.

[57] Kitaura H, Marahleh A, Ohori F, Noguchi T, Shen WR, Qi J, Nara Y, Pramusita A, Kinjo R, Mizoguchi I (2020). Osteocyte-related cytokines regulate osteoclast formation and bone resorption. Int J Mol Sci.

[58] Li R, Kato H, Nakata T, Yamawaki I, Yamauchi N, Imai K, Taguchi Y, Umeda M (2023). Essential amino acid starvation induces cell cycle arrest, autophagy, and inhibits osteogenic differentiation in murine osteoblast. Biochem Biophys Res Commun.

[59] Rong Y, Darnell AM, Sapp KM, Vander Heiden MG, Spencer SL (2023). Cells use multiple mechanisms for cell-cycle arrest upon withdrawal of individual amino acids. Cell Rep.

[60] Li R, Kato H, Fumimoto C, Nakamura Y, Yoshimura K, Minagawa E, Omatsu K, Ogata C, Taguchi Y, Umeda M (2023). Essential amino acid starvation-induced oxidative stress causes DNA damage and apoptosis in murine osteoblast-like cells. Int J Mol Sci.

[61] Shen L, Yu Y, Karner CM (2022). SLC38A2 provides proline and alanine to regulate postnatal bone mass accrual in mice. Front Physiol.

[62] Sharma D, Yu Y, Shen L, Zhang GF, Karner CM (2021). SLC1A5 provides glutamine and asparagine necessary for bone development in mice. Elife.

[63] Jiménez JA, Lawlor ER, Lyssiotis CA (2022). Amino acid metabolism in primary bone sarcomas. Front Oncol.

[64] Shen L, Sharma D, Yu Y, Long F, Karner CM (2021). Biphasic regulation of glutamine consumption by WNT during osteoblast differentiation. J Cell Sci.

[65] Nie C, He T, Zhang W, Zhang G, Ma X (2018). Branched chain amino acids: beyond nutrition metabolism. Int J Mol Sci.

[66] Brunner JS, Vulliard L, Hofmann M, Kieler M, Lercher A, Vogel A, Russier M, Brüggenthies JB, Kerndl M, Saferding V (2020). Environmental arginine controls multinuclear giant cell metabolism and formation. Nat Commun.

[67] Bordbar A, Mo ML, Nakayasu ES, Schrimpe-Rutledge AC, Kim YM, Metz TO, Jones MB, Frank BC, Smith RD, Peterson SN (2012). Model-driven multi-omic data analysis elucidates metabolic immunomodulators of macrophage activation. Mol Syst Biol.

[68] Onuora S (2023). L-arginine inhibits arthritis and bone loss by reprogramming osteoclast metabolism. Nat Rev Rheumatol.

[69] Shen Y, Wang H, Xie H, Zhang J, Ma Q, Wang S, Yuan P, Xue H, Hong H, Fan S (2024). l-arginine promotes angio-osteogenesis to enhance oxidative stress-inhibited bone formation by ameliorating mitophagy. J Orthop Translat.

[70] Stegen S, Moermans K, Stockmans I, Thienpont B, Carmeliet G (2024). The serine synthesis pathway drives osteoclast differentiation through epigenetic regulation of NFATc1 expression. Nat Metab.

[71] Zhou T, Yang Y, Chen Q, Xie L (2019). Glutamine metabolism is essential for stemness of bone marrow mesenchymal stem cells and bone homeostasis. Stem Cells Int.

[72] Yu Y, Newman H, Shen L, Sharma D, Hu G, Mirando AJ, Zhang H, Knudsen E, Zhang GF, Hilton MJ, Karner CM (2019). Glutamine metabolism regulates proliferation and lineage allocation in skeletal stem cells. Cell Metab.

[73] Gao P, Tchernyshyov I, Chang TC, Lee YS, Kita K, Ochi T, Zeller KI, De Marzo AM, Van Eyk JE, Mendell JT, Dang CV (2009). c-Myc suppression of miR-23a/b enhances mitochondrial glutaminase expression and glutamine metabolism. Nature.

[74] Tsumura H, Shindo M, Ito M, Igarashi A, Takeda K, Matsumoto K, Ohkura T, Miyado K, Sugiyama F, Umezawa A, Ito Y (2021). Relationships between Slc1a5 and osteoclastogenesis. Comp Med.

[75] Indo Y, Takeshita S, Ishii KA, Hoshii T, Aburatani H, Hirao A, Ikeda K (2013). Metabolic regulation of osteoclast differentiation and function. J Bone Miner Res.

[76] Peng R, Dong Y, Zheng M, Kang H, Wang P, Zhu M, Song K, Wu W, Li F (2024). IL-17 promotes osteoclast-induced bone loss by regulating glutamine-dependent energy metabolism. Cell Death Dis.

[77] Chen X, Wang Z, Duan N, Zhu G, Schwarz EM, Xie C (2018). Osteoblast-osteoclast interactions. Connect Tissue Res.

[78] Zhang W, Dang K, Huai Y, Qian A (2020). Osteoimmunology: The regulatory roles of T lymphocytes in osteoporosis. Front Endocrinol (Lausanne).

[79] Mellor AL, Munn DH (2004). IDO expression by dendritic cells: Tolerance and tryptophan catabolism. Nat Rev Immunol.

[80] Zara C, Severino A, Flego D, Ruggio A, Pedicino D, Giglio AF, Trotta F, Lucci C, D'Amario D, Vinci R (2017). Indoleamine 2,3-Dioxygenase (IDO) enzyme links innate immunity and altered T-cell differentiation in Non-ST segment elevation acute coronary syndrome. Int J Mol Sci.

[81] Eagle H, Oyama VI, Levy M, Horton CL, Fleischman R (1956). The growth response of mammalian cells in tissue culture to L-glutamine and L-glutamic acid. J Biol Chem.

[82] Colombo SL, Palacios-Callender M, Frakich N, Carcamo S, Kovacs I, Tudzarova S, Moncada S (2011). Molecular basis for the differential use of glucose and glutamine in cell proliferation as revealed by synchronized HeLa cells. Proc Natl Acad Sci USA.

[83] Ahn E, Kumar P, Mukha D, Tzur A, Shlomi T (2017). Temporal fluxomics reveals oscillations in TCA cycle flux throughout the mammalian cell cycle. Mol Syst Biol.

[84] Malakar P, Singha D, Choudhury D, Shukla S (2023). Glutamine regulates the cellular proliferation and cell cycle progression by modulating the mTOR mediated protein levels of β-TrCP. Cell Cycle.

[85] Minchenko DO, Hubenya OV, Terletsky BM, Moenner M, Minchenko OH (2011). Effect of glutamine or glucose deprivation on the expression of cyclin and cyclin-dependent kinase genes in glioma cell line U87 and its subline with suppressed activity of signaling enzyme of endoplasmic reticulum-nuclei-1. Ukr Biokhim Zh (1999).

[86] Yuan L, Sheng X, Willson AK, Roque DR, Stine JE, Guo H, Jones HM, Zhou C, Bae-Jump VL (2015). Glutamine promotes ovarian cancer cell proliferation through the mTOR/S6 pathway. Endocr Relat Cancer.

[87] Kim B, Li J, Jang C, Arany Z (2017). Glutamine fuels proliferation but not migration of endothelial cells. EMBO J.

[88] Chen Q, Shou P, Zheng C, Jiang M, Cao G, Yang Q, Cao J, Xie N, Velletri T, Zhang X (2016). Fate decision of mesenchymal stem cells: Adipocytes or osteoblasts?. Cell Death Differ.

[89] Ning K, Liu S, Yang B, Wang R, Man G, Wang DE, Xu H (2022). Update on the effects of energy metabolism in bone marrow mesenchymal stem cells differentiation. Mol Metab.

[90] Skerry TM (2008). The role of glutamate in the regulation of bone mass and architecture. J Musculoskelet Neuronal Interact.

[91] Wang Y, Deng P, Liu Y, Wu Y, Chen Y, Guo Y, Zhang S, Zheng X, Zhou L, Liu W (2020). Alpha-ketoglutarate ameliorates age-related osteoporosis via regulating histone methylations. Nat Commun.

[92] Fan M, Shi H, Yao H, Wang W, Zhang Y, Jiang C, Lin R (2022). Glutamate regulates gliosis of BMSCs to promote ENS regeneration through α-KG and H3K9/H3K27 demethylation. Stem Cell Res Ther.

[93] Qian D, Wei G, Xu C, He Z, Hua J, Li J, Hu Q, Lin S, Gong J, Meng H (2017). Bone marrow-derived mesenchymal stem cells (BMSCs) repair acute necrotized pancreatitis by secreting microRNA-9 to target the NF-κB1/p50 gene in rats. Sci Rep.

[94] Ganesan R, Rasool M (2017). Interleukin 17 regulates SHP-2 and IL-17RA/STAT-3 dependent Cyr61, IL-23 and GM-CSF expression and RANKL mediated osteoclastogenesis by fibroblast-like synoviocytes in rheumatoid arthritis. Mol Immunol.

[95] Saraiva M, Vieira P, O'Garra A (2020). Biology and therapeutic potential of interleukin-10. J Exp Med.

[96] Mielle J, Morel J, Elhmioui J, Combe B, Macia L, Dardalhon V, Taylor N, Audo R, Daien C (2022). Glutamine promotes the generation of B10+ cells via the mTOR/GSK3 pathway. Eur J Immunol.

[97] Liu JQ, Geng XR, Hu TY, Mo LH, Luo XQ, Qiu SY, Liu DB, Liu ZG, Shao JB, Liu ZQ, Yang PC (2022). Glutaminolysis is required in maintaining immune regulatory functions in B cells. Mucosal Immunol.

[98] Coëffier M, Marion R, Ducrotté P, Déchelotte P (2003). Modulating effect of glutamine on IL-1beta-induced cytokine production by human gut. Clin Nutr.

[99] Santos AC, Correia CA, de Oliveira DC, Nogueira-Pedro A, Borelli P, Fock RA (2016). Intravenous glutamine administration modulates TNF-α/IL-10 ratio and attenuates NFkB phosphorylation in a protein malnutrition model. Inflammation.

[100] da Silva Lima F, Rogero MM, Ramos MC, Borelli P, Fock RA (2013). Modulation of the nuclear factor-kappa B (NF-κB) signalling pathway by glutamine in peritoneal macrophages of a murine model of protein malnutrition. Eur J Nutr.

[101] Sun Y, Ma J, Li D, Li P, Zhou X, Li Y, He Z, Qin L, Liang L, Luo X (2019). Interleukin-10 inhibits interleukin-1β production and inflammasome activation of microglia in epileptic seizures. J Neuroinflammation.

[102] Levy DE, Lee CK (2002). What does Stat3 do?. J Clin Invest.

[103] Dos Santos GG, Hastreiter AA, Sartori T, Borelli P, Fock RA (2017). L-Glutamine in vitro modulates some immunomodulatory properties of bone marrow mesenchymal stem cells. Stem Cell Rev Rep.

[104] Refaey ME, McGee-Lawrence ME, Fulzele S, Kennedy EJ, Bollag WB, Elsalanty M, Zhong Q, Ding KH, Bendzunas NG, Shi XM (2017). Kynurenine, a tryptophan metabolite that accumulates with age, induces bone loss. J Bone Miner Res.

[105] Dalton S, Smith K, Singh K, Kaiser H, Kolhe R, Mondal AK, Khayrullin A, Isales CM, Hamrick MW, Hill WD, Fulzele S (2020). Accumulation of kynurenine elevates oxidative stress and alters microRNA profile in human bone marrow stromal cells. Exp Gerontol.

[106] Sas K, Szabó E, Vécsei L (2018). Mitochondria, oxidative stress and the kynurenine system, with a focus on ageing and neuroprotection. Molecules.

[107] Elmansi AM, Hussein KA, Herrero SM, Periyasamy-Thandavan S, Aguilar-Pérez A, Kondrikova G, Kondrikov D, Eisa NH, Pierce JL, Kaiser H (2020). Age-related increase of kynurenine enhances miR29b-1-5p to decrease both CXCL12 signaling and the epigenetic enzyme Hdac3 in bone marrow stromal cells. Bone Rep.

[108] Kondrikov D, Elmansi A, Bragg RT, Mobley T, Barrett T, Eisa N, Kondrikova G, Schoeinlein P, Aguilar-Perez A, Shi XM (2020). Kynurenine inhibits autophagy and promotes senescence in aged bone marrow mesenchymal stem cells through the aryl hydrocarbon receptor pathway. Exp Gerontol.

[109] Anaya JM, Bollag WB, Hamrick MW, Isales CM (2020). The role of tryptophan metabolites in musculoskeletal stem cell aging. Int J Mol Sci.

[110] Sautchuk R, Eliseev RA (2022). Cell energy metabolism and bone formation. Bone Rep.

[111] Li S, Tian Q, Zheng L, Zhou Y (2024). Functional amino acids in the regulation of bone and its diseases. Mol Nutr Food Res.

[112] Ledesma-Colunga MG, Passin V, Lademann F, Hofbauer LC, Rauner M (2023). Novel insights into osteoclast energy metabolism. Curr Osteoporos Rep.

[113] Carbone L, Bůžková P, Fink HA, Robbins JA, Barzilay JI, Elam RE, Isales C, Connelly MA, Mukamal KJ (2023). Plasma levels of branched chain amino acids, incident hip fractures, and bone mineral density of the hip and spine. J Clin Endocrinol Metab.

[114] Su Y, Elshorbagy A, Turner C, Refsum H, Chan R, Kwok T (2019). Circulating amino acids are associated with bone mineral density decline and ten-year major osteoporotic fracture risk in older community-dwelling adults. Bone.

[115] Liang B, Shi X, Wang X, Ma C, Leslie WD, Lix LM, Shi X, Kan B, Yang S (2024). Association between amino acids and recent osteoporotic fracture: A matched incident case-control study. Front Nutr.

[116] Zhang YY, Xie N, Sun XD, Nice EC, Liou YC, Huang C, Zhu H, Shen Z (2024). Insights and implications of sexual dimorphism in osteoporosis. Bone Res.

[117] Guan Z, Luo L, Liu S, Guan Z, Zhang Q, Li X, Tao K (2022). The role of depletion of gut microbiota in osteoporosis and osteoarthritis: A narrative review. Front Endocrinol (Lausanne).

[118] Hao L, Yan Y, Huang G, Li H (2024). From gut to bone: deciphering the impact of gut microbiota on osteoporosis pathogenesis and management. Front Cell Infect Microbiol.

